# Identifying the natural products in the treatment of atherosclerosis by increasing HDL-C level based on bioinformatics analysis, molecular docking, and in vitro experiment

**DOI:** 10.1186/s12967-023-04755-7

**Published:** 2023-12-19

**Authors:** Yilin Chen, Fengwei Zhang, Jijia Sun, Lei Zhang

**Affiliations:** 1 Shanghai Innovation Center of Traditional Chinese Medicine Health Service, Shanghai University of Traditional Chinese Medicine, Shanghai, China; 2 Department of Mathematics and Physics, School of Pharmacy, Shanghai University of Traditional Chinese Medicine, Shanghai, China

**Keywords:** Atherosclerosis, High-density lipoprotein cholesterol, Differential gene analysis, PPI network analysis, Molecular docking, Genistein

## Abstract

**Background:**

Previous studies have demonstrated that high-density lipoprotein cholesterol (HDL-C) plays an anti-atherosclerosis role through reverse cholesterol transport. Several studies have validated the efficacy and safety of natural products in treating atherosclerosis (AS). However, the study of raising HDL-C levels through natural products to treat AS still needs to be explored.

**Methods:**

The gene sets associated with AS were collected and identified by differential gene analysis and database query. By constructing a protein–protein interaction (PPI) network, the core submodules in the network are screened out. At the same time, by calculating node importance (Nim) in the PPI network of AS disease and combining it with Kyoto Encyclopedia of genes and genomes (KEGG) pathways enrichment analysis, the key target proteins of AS were obtained. Molecular docking is used to screen out small natural drug molecules with potential therapeutic effects. By constructing an in vitro foam cell model, the effects of small molecules on lipid metabolism and key target expression of foam cells were investigated.

**Results:**

By differential gene analysis, 451 differential genes were obtained, and a total of 313 disease genes were obtained from 6 kind of databases, then 758 AS-related genes were obtained. The enrichment analysis of the KEGG pathway showed that the enhancement of HDL-C level against AS was related to Lipid and atherosclerosis, Cholesterol metabolism, Fluid shear stress and atherosclerosis, PPAR signaling pathway, and other pathways. Then we intersected 31 genes in the core module of the PPI network, the top 30 genes in Nims, and 32 genes in the cholesterol metabolism pathway, and finally found 3 genes. After the above analysis and literature collection, we focused on the following three related gene targets: *APOA1*, *LIPC*, and *CETP*. Molecular docking showed that Genistein has a good binding affinity for *APOA1*, *CETP*, and *LIPC*. In vitro, experiments showed that Genistein can up-regulated *APOA1*, *LIPC*, and *CETP* levels.

**Conclusions:**

Based on our research, Genistein may have the effects of regulating HDL-C and anti-atherosclerosis. Its mechanism of action may be related to the regulation of *LIPC*, *CETP*, and *APOA1* to improve lipid metabolism.

**Supplementary Information:**

The online version contains supplementary material available at 10.1186/s12967-023-04755-7.

## Background

Atherosclerosis (AS) is a chronic immunoinflammatory disease caused by an imbalance in the metabolism of lipids, which leads to long-lasting damage to the body’s immune system. Atherosclerotic plaque is formed when cholesterol, fat, calcium, and other substances are deposited on the inner wall of blood vessels. It narrows blood vessels and reduces blood flow and velocity, leading to insufficient blood supply to local tissues [1]. Carotid atherosclerosis is the manifestation of AS in the carotid arteries and is a common site in the evaluation of clinical AS [2]. According to the World Health Organization report, AS is the leading cause of death in both developed and developing countries. With the rapid growth of the global economy and the widespread popularity of Western diets, the mortality and prevalence of AS in contemporary society are rising. In recent years, AS has shown a high incidence and is the common cause of death [3]. AS is the main underlying factor in many cardiovascular diseases (CVDs) and remains the leading cause of death worldwide. Previous studies have demonstrated that increasing functional high-density lipoprotein (HDL) levels in people at risk of CVD events may be a feasible therapy to inhibit AS progression and promote AS regression [4]. The exploration of emerging therapeutic approaches for AS has been a hot topic in recent years, such as magnetic nanoparticles, which can be used for magnetic drug targeting. In recent years, there have been several articles on the study of the phenomenon of biomineralization and the production of inorganic magnetic nanoparticles in biological systems. The green synthesis method used in the synthesis of nanoparticles is currently the main area of interest for researchers [5, 6].

Many epidemiological studies have identified an inverse correlation between HDL levels and AS [7, 8]. HDL is protective because it removes excess cholesterol from surrounding tissues by reverse cholesterol transport (RCT) and transporting it to the liver for bile excretion [9]. In addition, low high-density lipoprotein cholesterol (HDL-C) levels increase beta cell dysfunction and aggravate insulin resistance. Furthermore, long-term high insulin levels may promote damage to the lining of the artery wall and promote plaque formation. In addition, insulin resistance is also associated with other risk factors, such as high blood pressure, high blood lipids, and obesity, which are also risk factors for AS [10]. Despite this, no drugs have been found with a definite effect of raising HDL-C, including chemical drugs and natural products to date.

Natural products are the components or metabolites of living organisms that have evolved in nature over a long period. For example, flavonoid compounds present in plants and polysaccharides in plant cell walls show a variety of pharmacological effects. Therefore, it is seen as a potential alternative to traditional treatment methods in the future [11]. In particular, the effective ingredients in Traditional Chinese Medicine (TCM), which uphold thousands of years of history and clinical practice, are widely used in the prevention and treatment of diseases [12]. The efficacy and safety of TCM have been widely recognized in TCM theory and clinical practice, but to ensure its rational use, modern TCM research also pays attention to scientific research and clinical trials on the effective ingredients, pharmacological effects, and pharmacokinetics of TCM [13]. TCM has a long history in the treatment of AS and rich experience in clinical application. AS was documented thousands of years ago in China and was treated with Chinese herbal medicine [14]. In TCM theory, AS is often referred to as “MaiBi”, a vascular problem caused by *Qi* stagnation, *Blood* stasis, and/or *Phlegm* coagulation [15]. Currently, a variety of chemical drugs have been used to treat AS, but their mechanism of action is not clear [16, 17], some of them have poor efficacy and serious side effects [18]. It is very important to develop safe and efficient AS treatment drugs. TCM has potential use in the treatment of AS because of their few toxic and side effects, strong safety, definite curative effect, and economic benefits [19].

In recent years, the rise of bioinformatics has undoubtedly provided a new analysis method for exploring the mechanism of multi-target and multi-pathway diseases and the pharmacological effects of drugs [20]. A network pharmacological research method has been proposed by pharmacological and systems biology research tools to predict the underlying mechanism of TCM efficacy [21]. It breaks the traditional thinking mode of “one disease-one target-one drug” and reveals the etiological mechanism of complex diseases in a multi-angle and multi-approach way [22]. It can then be used in conjunction with molecular docking to aid drug discovery. It has emerging uses and applications, including adverse reaction prediction, multi-pharmacology, and drug reuse [23].

Although several TCM compounds have been reported to have good therapeutic effects and mild side effects, it is of great practical significance to find additional potential anti-AS components from a large number of common TCM small molecules with clear pharmacological actions. Given this, we combined bioinformatics, computer-aided drug design, and analysis of existing literature, to explore important targets for the treatment of AS, and further screen out effective components of TCM with potential therapeutic effects. This study further verified the above conclusions through in vitro experiments, and the potential natural product was found to improve the level of HDL-C by regulating multiple targets and pathways, playing an anti-AS role, and providing powerful methods and technical support for the treatment of AS by natural products.

**Fig. 1:**
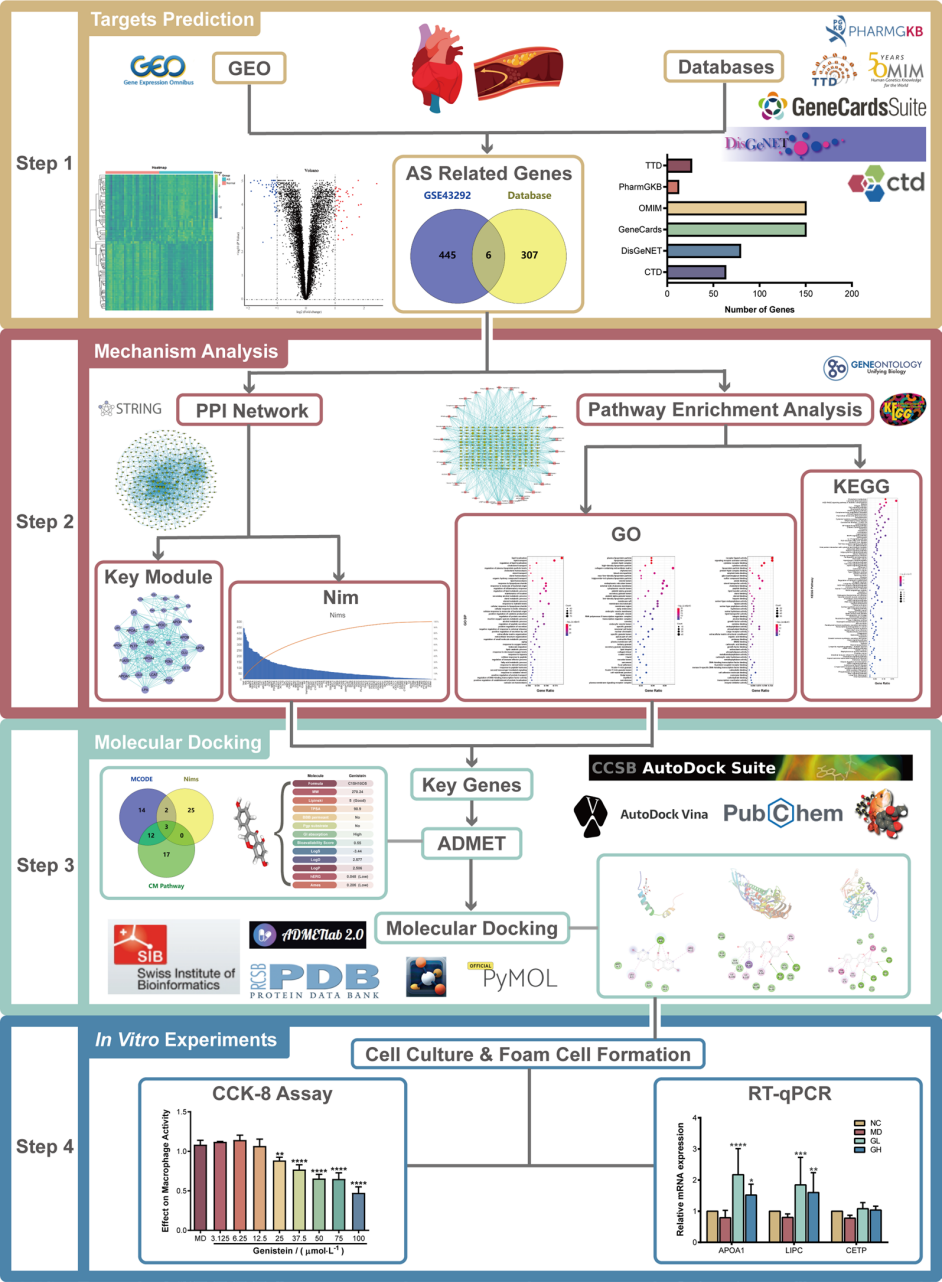
Flowchart of the study of potential natural products for the treatment of AS by elevating HDL-C levels based on bioinformatics analysis

The flowchart of the present study is illustrated in Fig. 1.

## Materials and methods

### Therapeutic targets for AS

In the Gene Expression Omnibus (GEO) database, input the “Atherosclerosis” query and collection of AS related Gene Expression profile chip data. R (version 4.1.2) was used to perform preprocessing of the original data, such as standardization, correction, and gene name annotation. The R language-based limma package [24] (version 3.44.0) was used for differentially expressed genes (DEGs) analysis. The upregulated and downregulated DEGs in each set of chip data were screened out when log2|Fold Change| > 1.0 and *P*-value < 0.05.

To ensure the integrity of the collected AS-related gene set, according to our previous research experience [25, 26], six commonly used databases were queried and collected, namely: CTD [27], DisGeNET [28], GeneCards [29], OMIM [30, 31], PharmGKB [32], and TTD [33]. The keyword “Atherosclerosis” was input into 6 databases to query and collect genes associated with AS disease. CTD database to select the Direct Evidence tag “marker/mechanism”, “marker/mechanism | therapeutic” and “therapeutic” genes. The gene with Score_gda > 0.1 was selected in DisGeNET. Genes with a Relevance score > 5 were selected in GeneCards. OMIM selected genes with a definite Entrez Gene ID. In PharmGKB and TTD databases, all relevant genes were included in the AS-related gene set by inputting keywords.

Then, the results of differential gene analysis and screening based on GEO were combined with the target genes queried from 6 databases to remove duplicate genes. All the collected target information was confirmed through the UniProt to form an AS disease-related target gene collection S_AS_. It should be noted that the deadline for our query and use of all the above databases is September 2022.

### Construction of the protein–protein interaction (PPI) network

All genes obtained above were imported into STRING (version 11.0) [34] to obtain the PPI network of AS disease. Here, the specific parameter is set: Organism is “Homo Sapiens”, a combined score > 0.9. Meanwhile, we used Gephi software (version 0.9.2) to visualize the network. In addition, the molecular complex detection (MCODE) [35] tool in Cytoscape software (version 3.7.1) [36] was used to identify the key modules in the PPI network, where the parameters were set to default values.

Node importance (Nim) is an important topological property and can be used to evaluate the influence of nodes among the network. The nodes whose Nim is larger than the average Nim of all nodes are treated as critical roles and hub nodes in the network. We carried out optimization based on literature and used equation [37] to calculate Nim in AS disease PPI network:

1 $${\text{Nim}}_{(s)}=\sqrt{\left[\sum _{s\ne v\ne t\in V}\frac{{\sigma }_{vt}(s)}{{\sigma }_{vt}}\right]\times \sum _{s\ne x}\exp\left[-d(s,x)\right]}$$

Among them, Nim is the importance value of each node; ${\sigma }_{vt}$ is the number of shortest paths between node $v$ and nod $t$; ${{\sigma }_{vt}}(s)$is the number of shortest paths between node $v$ and nod $t$ passing through node $s$; And $d(s,x)$ is the shortest path distance between nodes $s$ and all connection points in the network. It is achieved by using the igraph package (version 1.2.6).

### Gene ontology (GO) function and Kyoto Encyclopedia of genes and genomes (KEGG) pathway enrichment analysis

The GO database is a structured standard biological model constructed by the GO Consortium in 2000. It covers the Biological Process (BP), Molecular Function (MF), and Cellular Component (CC) of genes [38]. A biological process or pathway is usually performed by a group of genes working together, rather than by a single gene. The main basis of enrichment analysis is that if a biological process or pathway is abnormal in known studies, the genes that function together are most likely to be selected as the gene set associated with this process or pathway. In this study, we further used the clusterProfiler (version 3.14.3) [39] toolkit to conduct GO function and KEGG Pathway enrichment analysis on the target set of potential AS diseases and screened them according to *P*-value < 0.05 and *Q*-value < 0.05. The gene-pathway network interrelationships are also mapped using Cytoscape.

### Screening of small molecules on key targets and molecular docking

To screen potential natural products, we use molecular docking techniques to examine the affinity between the receptor and the ligand. The selected key protein targets met the following conditions: (1) The pathway closely related to AS disease in the KEGG pathway enrichment analysis results; (2) The target was a key protein target in the PPI network; (3) In the Nims calculation results. After selecting potential key targets for the treatment of AS diseases, the PDB files of potential key target proteins is downloaded from the PDB database [40]. PyMOL software (version 1.7.0) pretreats key target proteins to remove other miscellaneous ions, including water molecules. We searched the HIT2.0 [41] for potentially active small molecules of the target protein. The small molecules downloaded from PubChem are available in two SDF formats, 2D and 3D. 2D SDF files are made use of OpenBabel software (version2.4.0) to a 3D structure file. The original 3D structure is converted directly to a PDB file. AutoDock Tools (version 1.5.6) was used to hydrogenate the treated protein ligands, charge them, transform them, and save them as PDBQT files. The docking center parameters were determined by referring to the binding site (region) of the protein receptor and the original ligand. Define the box size to be 30 × 30 × 30. Use AutoDock Vina (version 1.1.2) software for semi-flexible molecular docking. The affinity (kcal/mol) between all small molecules and potential key targets was calculated. The lower the affinity value, the more stable the interaction between the target protein and the active ingredient. Finally, small molecules that potentially treat AS by increasing HDL-C levels were selected according to the affinity values from low to high.

### Cell culture

The mouse macrophage cell line RAW264.7 (Simuwu Bio, Shanghai, China) was maintained in Dulbecco’s modified Eagle’s medium (DMEM, Gibco, Newyork, USA) supplemented with 10% FBS. RAW264.7 cells at the logarithmic growth stage were inoculated into 6-well plates, each well 5 × 10^5^. When the cells were fully adherent, they were transformed into foam cells by incubation for 24 h with DMEM supplemented with 10% FBS containing oxidized low-density lipoprotein (ox-LDL, Yiyuan Biotechnologies, Guangzhou, China) (50 µg/mL).

### CCK-8 assay

RAW264.7 cells were inoculated into 96-well plates with 4 × 10^3^ cells per well. Add 100 µL (3.125, 6.25, 12.5, 25, 37.5, 50, 75, 100 µmol/L) Genistein (Yuanye, Shanghai, China) to each well. The control group was added with DMEM complete medium. After 24 h culture, 10 µL CCK-8 solution was added to each well. The culture was continued in the incubator for 2 h. The absorbance at 450 nm was determined by an enzyme-labeled instrument.

### Reverse transcription‑quantitative polymerase chain reaction (RT‑qPCR)

Total RNA was extracted from cells by RNA-Easy Isolation Reagent (Vazyme, Nanjing, China). And for real-time quantitative PCR analysis, cDNA was synthesized using HiScript II Q RT SuperMix for qPCR Kit (Vazyme, Nanjing, China). We then used SYBR Green dye combined with *APOA1*, *LIPC*, and *CETP* primer pairs for mRNA quantification. All primers were purchased from Sangon Biotech (Shanghai, China). The primer sequence is shown in Additional file 1: Table S1. And the relative mRNA expression was normalized with GAPDH using the ΔΔCt method. 0.1% MDSO (Adamas, Shanghai, China) was added to the DMEM of the NC group. In the MD group, 10% FBS containing ox-LDL (50 µg/mL) was added to DMEM as a culture system. In the treatment group, 10% ox-LDL (50 µg/mL), FBS, and corresponding small molecules of TCM were added into DMEM, and the cells were transformed into foam cells after incubation for 24 h.

### Statistical analysis

Data from RT-qPCR experiments were plotted and analyzed by GraphPad Prism 9.0 and SPSS 26.0. Comparisons among multiple groups were performed using one-way analysis of variance (ANOVA) for normal distribution and Kruskal–Wallis test for non-normal distribution. One-way ANOVA was followed by a least significant difference (LSD) test. Comparisons between the two groups were performed using an unpaired t-test for normal distribution and Mann–Whitney test for non-normal distribution. *P*-value < 0.05 was considered statistically significant.

## Results

### AS chip data collection and differential gene analysis

**Fig. 2:**
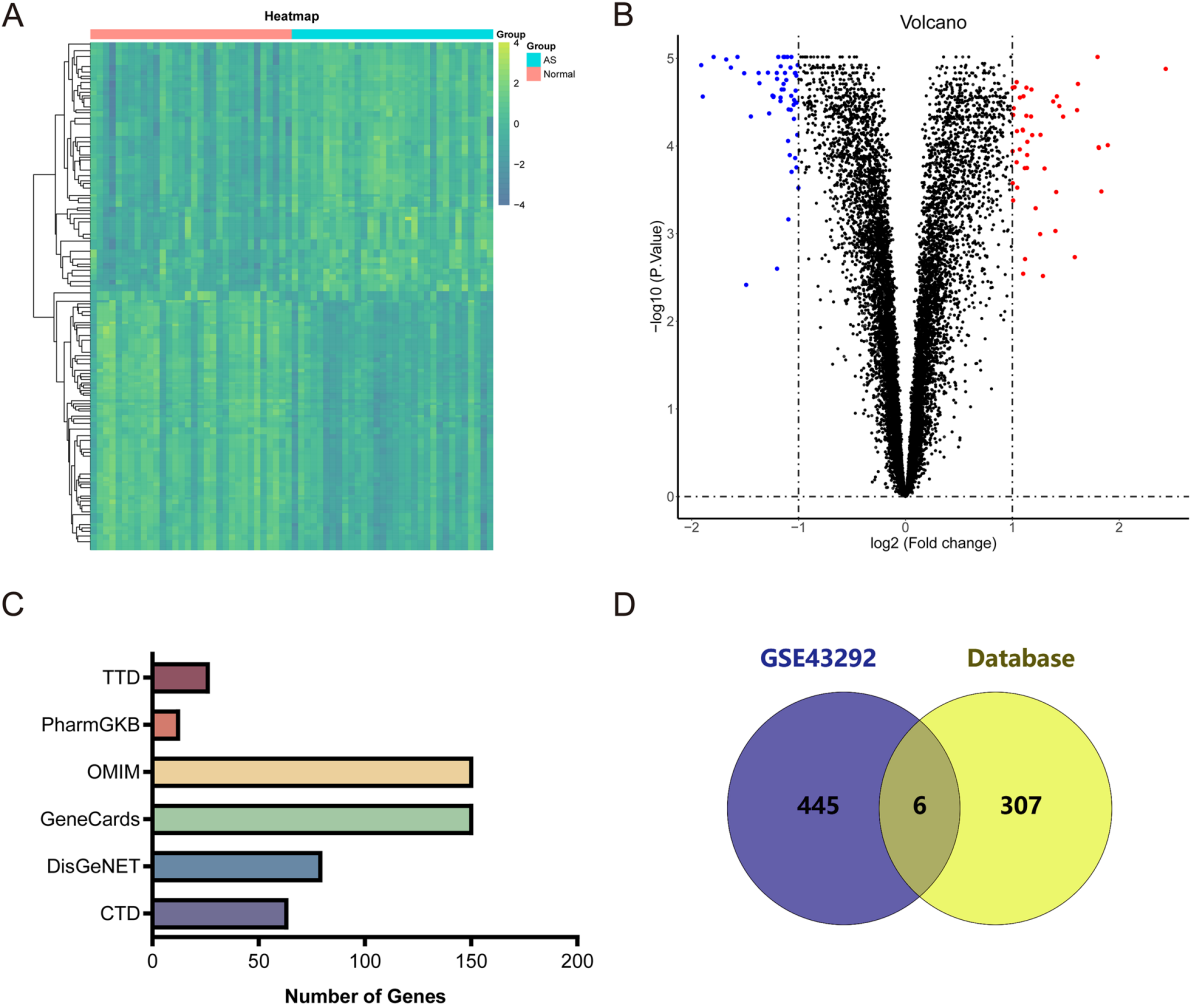
AS chip data collection and differential gene analysis. **A** Heat map of GSE43292 differential gene analysis results. **B** Volcanic map of GSE43292 differential gene analysis results. **C** Gene distribution in different drug target databases. **D** AS target gene collection based on GEO differential gene analysis and drug target database

GSE43292 was obtained from the GEO query to obtain the expression profile data related to AS. The platform of GSE43292 is [HuGene-1_0-st] Affymetrix Human Gene 1.0 ST Array [transcript (gene) version]. There were 32 normal samples and 32 AS samples. After analysis, 451 differential genes were screened from GSE43292. There were 221 up-regulated genes and 230 down-regulated genes (Fig. 2A, B). As shown in Fig. 2C, 64, 80, 151, 151, 13, and 27 AS targets were screened from CTD, DisGeNET, GeneCards, OMIM, PharmGKB, and TTD databases. A total of 313 genes related to AS were obtained after consolidation. Finally, all genes obtained by the above two methods (based on differential gene analysis and target database collection) were combined. A total of 758 target genes related to AS were obtained, namely the target gene set S_AS_ (Fig. 2D).

### PPI network construction and screening of key targets

**Fig. 3:**
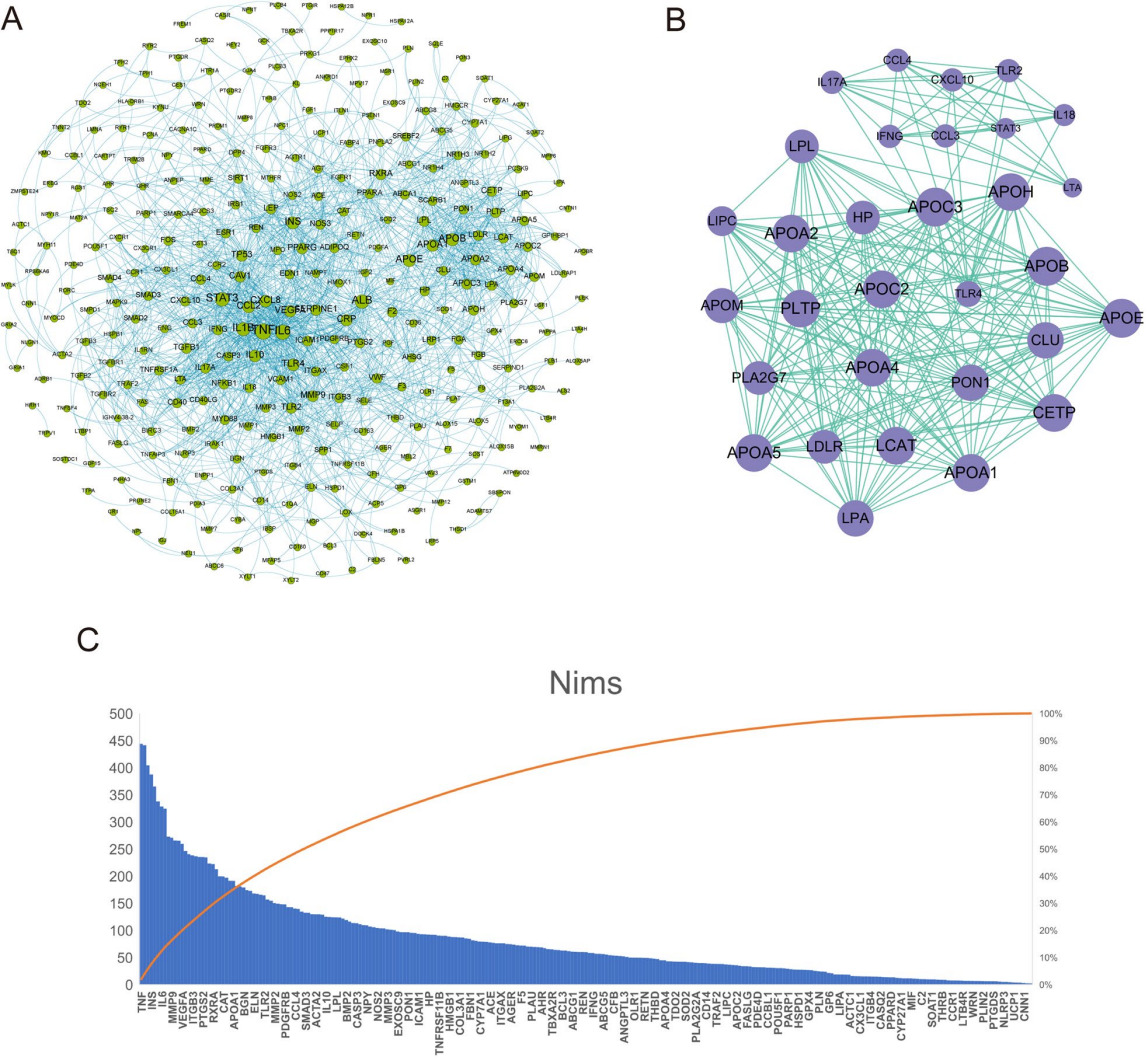
PPI network construction and screening of key targets. **A** AS disease PPI network constructed by differential gene analysis and STRING database, in which dots represent proteins, and the larger the nodal degree value is. **B** Top 31 important proteins and their core submodules in the PPI network obtained based on the MCC algorithm. **C** Node importance (Nim)

All 758 AS differential genes were imported into the STRING database. Therefore, the corresponding PPI network is obtained (Fig. 3A). Through PPI network module analysis, we screened out a core submodule. They are *APOA1*, *APOA2*, *APOA4*, *APOA5*, *APOB*, *APOC2*, *APOC3*, *APOE*, *APOH*, *APOM*, *CCL3*, *CCL4*, *CETP*, *CLU*, *CXCL10*, *HP*, *IFNG*, *IL17A*, *IL18*, *LCAT*, *LDLR*, *LIPC*, *LPA*, *LPL*, *LTA*, *PLA2G7*, *PLTP*, *PON1*, *STAT3*, *TLR2*, and *TLR4*. These 31 important proteins become a highly interconnected submodule (Fig. 3B).

We use formula to calculate Nim in the AS disease PPI network (Fig. 3C). We selected the top 30 nodes for path enrichment. Fifteen of them were enriched in the Cholesterol metabolism pathway.

### GO function annotation and KEGG pathway enrichment analysis results

**Fig. 4:**
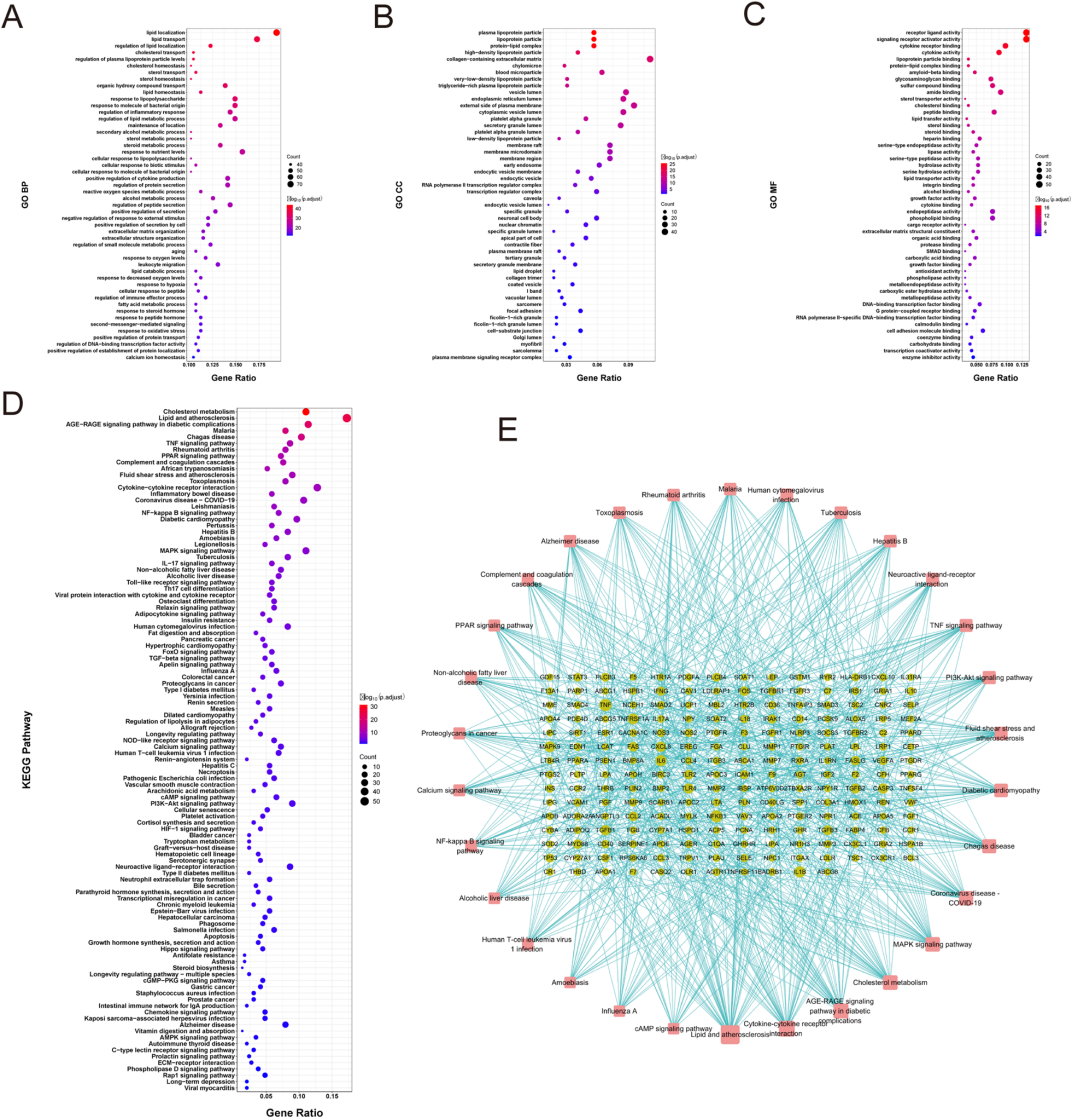
GO function annotation and KEGG pathway enrichment analysis results. **A**–**C** GO functional annotation of AS differential genes (BP, CC and MF). **D** KEGG pathway enrichment analysis of AS differential genes. **E** AS target-pathway enrichment network, with dots representing protein targets and squares representing pathways. The line between the protein target and the pathway indicates that there is an enrichment relationship between the target protein and a certain pathway

ClusterProfiler is used to further GO functional and KEGG Pathway enrichment analysis on 734 differential gene sets of AS, where *P*-value < 0.05 and *Q*-value < 0.05. The GO function annotation results of the target show that there are 2371 BP functions involved, such as lipid localization (GO:0010876), lipid transport (GO:0006869), regulation of lipid localization (GO:1,905,952), cholesterol transport (GO:0030301), regulation of plasma lipoprotein particle levels (GO:0097006) (Fig. 4A); There are 54 CC functions involved, such as plasma lipoprotein particle (GO:0034358), lipoprotein particle (GO:1990777), protein–lipid complex (GO:0032994), high-density lipoprotein particle (GO:0034364), collagen-containing extracellular matrix (GO:0062023), among several others (Fig. 4B); And there are 158 MF functions involved, such as receptor-ligand activity (GO:0048018), signaling receptor activator activity (GO:0030546), cytokine receptor binding (GO:0005126), amide binding (GO:0033218) (Fig. 4C). KEGG pathway enrichment analysis showed that the target was mainly enriched in 108 related signaling pathways. They are Lipid and atherosclerosis (hsa05417), Cholesterol metabolism (hsa04979), Fluid shear stress and atherosclerosis (hsa05418), and PPAR signaling pathway (hsa03320). The above pathways have been reported to be closely related to AS disease (Fig. 4D). The gene-enrichment pathway network is shown in Fig. 4E.

**Fig. 5:**
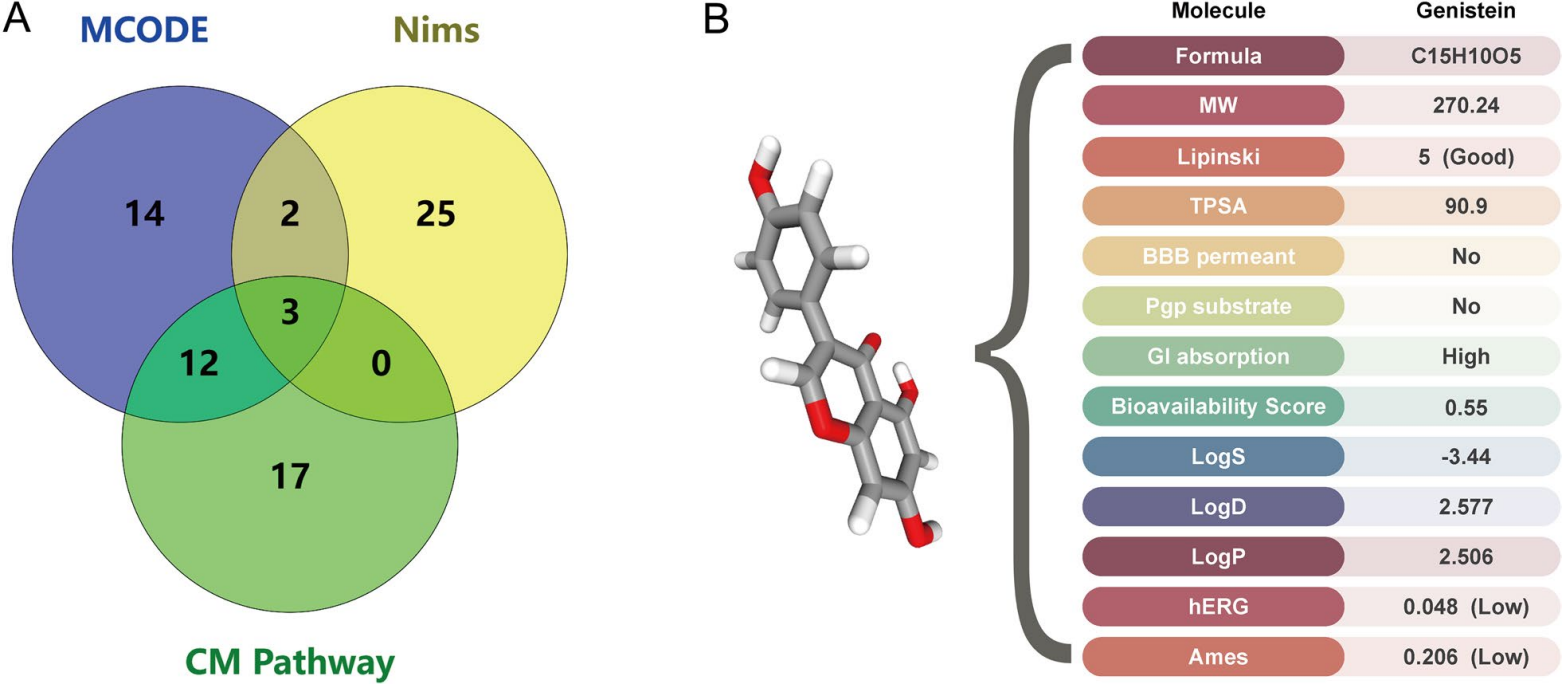
Identification of target genes and potential small molecules. **A** Target gene collection based on the module, the Nims, and the Cholesterol metabolism pathway. **B** ADMET of Genistein

After the intersection of the gene sets obtained by the above three methods of PPI network module analysis, Nims calculation results, and KEGG pathway enrichment analysis, we found that three genes (*APOA1*, *APOB*, *APOE*) simultaneously met the above three conditions. Based on a literature review, we are particularly concerned about Apolipoprotein A1 (*APOA1*) (Fig. 5a).

### Molecular docking analysis

In this study, we used HIT 2.0 to search for potential TCM small molecules that interact with a key target protein, *APOA1*. The results showed that 6 small molecules met the conditions（Additional file 2: Table S2.). According to the literature research [42, 43], we selected Genistein. ADMET of Genistein was analyzed by ADMETlab 2.0 [44] and SwissADME [45] respectively, as shown in Fig. 5b. The results showed that the small molecule had good drug properties and was a good potential therapeutic compound.

**Fig. 6:**
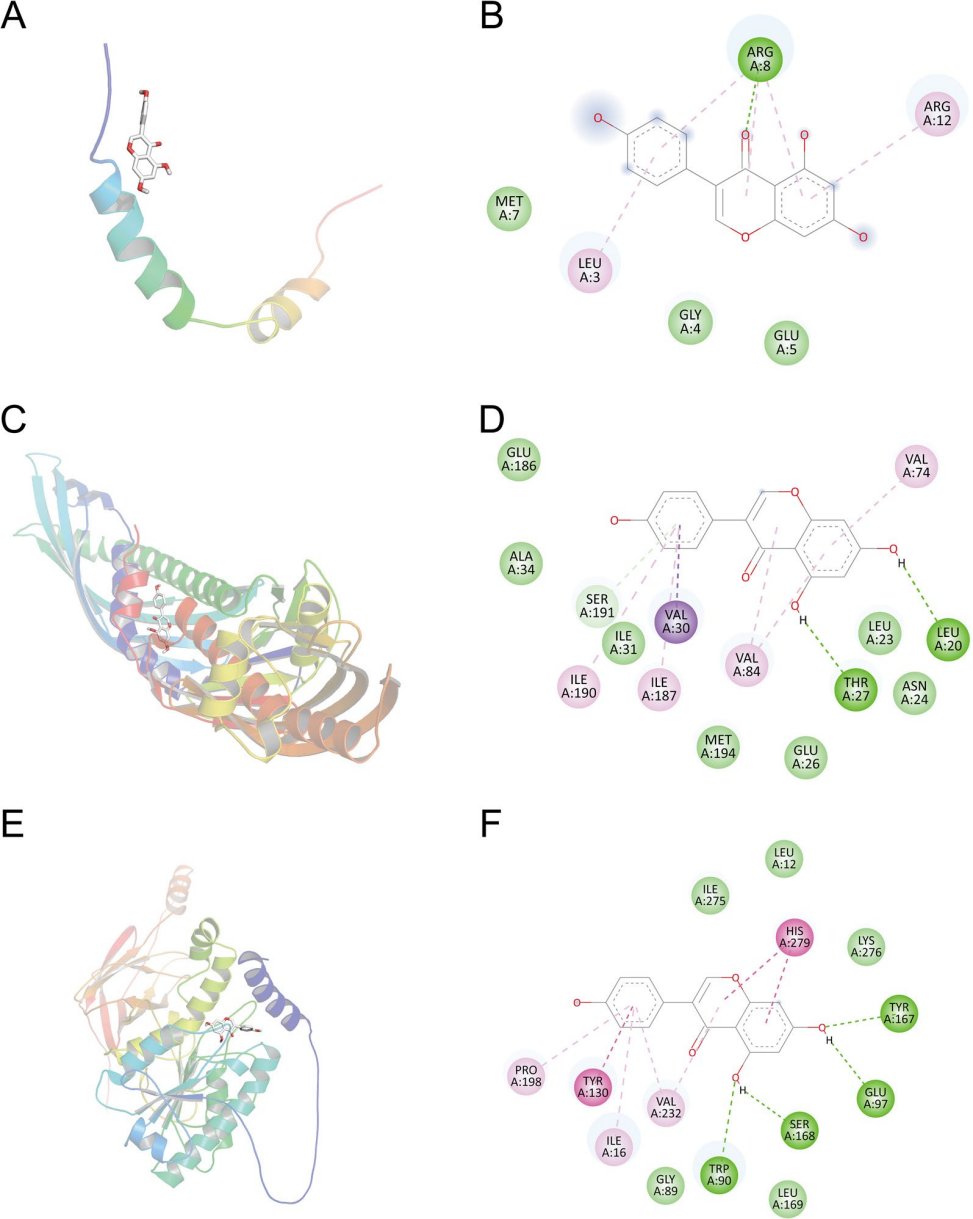
Molecular docking results of Genistein interaction with *APOA1*, *LIPC*, and *CETP*. **A**, **B** Molecular docking conformation of Genistein interaction with *APOA1*. **C**, **D** Molecular docking conformation of Genistein interaction with *LIPC*. **E**, **F** Molecular docking conformation of Genistein interaction with *CETP*

In addition to *APOA1*, we also selected *LIPC* and *CETP*, which are related to AS, from the PPI network module. We found that Genistein’s affinity values for *LIPC*, *APOA1*, and *CETP* were − 9.5, − 7.7, and − 5.1 (Table 1). Under normal circumstances, the binding energy is less than − 5, which means that it has good binding potential. The docking modes between Genistein and the three targets are shown in Fig. 6A, C, E. The 2D binding conformations of Genistein and target proteins were displayed using BIOVIA Discovery Studio Visualizer, as shown in Fig. 6B, D, F.

**Table 1:** Molecular docking results of AS with target proteins

MOL	Targets	Pathway	Protein names	Uniprot ID	PDB ID	Affinity (kcal/mol)
Genistein	*APOA1*	Cholesterol metabolism	Apolipoprotein A-I	P02647	1GW3	− 5.1
Genistein	*CETP*	Cholesterol metabolism	Cholesteryl ester transfer protein	P11597	4F2A	− 7.7
Genistein	*LIPC*	Cholesterol metabolism	Hepatic triacylglycerol lipase	P11150	AlphaFold	− 9.5

### Verification with in vitro cell culture experiments

**Fig. 7:**
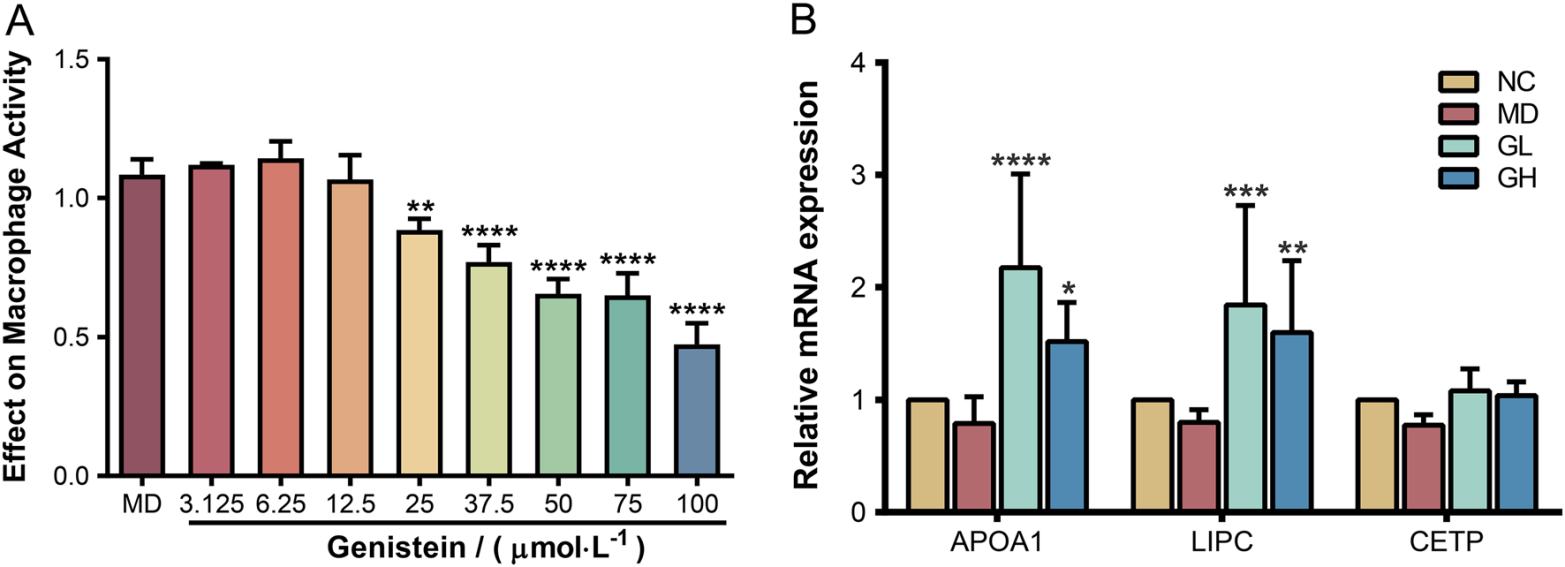
Verification with in vitro cell culture experiments. **A** Effect of Genistein on the viability of macrophage $({\bar{\text{x}}} \pm {\text{s}})$. **B** The relative *APOA1*, *LIPC*, and *CETP* mRNA expression levels. *P < 0.05, **P < 0.01, ***P < 0.001, ****P < 0.0001

Based on the results of molecular docking, Genistein was selected for in vitro experiments. The results of CCK-8 showed that the level of cell proliferation was not significantly affected when the concentration of Genistein was below 12.5 µmol/L (Fig. 7A). According to the safe dose range, we selected the high dose of Genistein as 10 µmol/L and the low dose as 2.5 µmol/L in the follow-up experiment.

The mRNA expression levels of target proteins in the NC, MD, and treated groups were measured by RT-qPCR. The RT-qPCR results (Fig. 7B) showed that the mRNA expression levels of *APOA1*, *LIPC*, and *CETP* in the MD group were lower than those in the NC group. Both low and high doses of Genistein could increase the relative expression of *APOA1*, *LIPC*, and *CETP*. Notably, low-dose Genistein therapy reversed the reduction in mRNA expression of target proteins induced by ox-LDL intervention and improved cellular fat metabolism. However, the regulation of *CETP* by Genistein was not obvious.

## Discussion

HDL-C is considered the “good” cholesterol. Pharmacoepidemiological studies have shown that HDL-C levels are inversely associated with the incidence of CVD [7, 46]. For a long time, the prevention and treatment of AS by increasing HDL-C has been considered to have broad prospects. However, the drugs currently screened for raising HDL-C do not play this role well [47]. For example, niacin can increase HDL-C levels, but studies have determined that niacin does not reduce residual cardiovascular risk [48]. *CETP* blockers have been eliminated because they have more adverse reactions or are not beneficial to the end event [49, 50]. The *PPARα* agonist K-877 is in Phase II clinical trials [51]. Based on the above studies, we believe that actively exploring natural products to improve HDL-C has important clinical value.

In this study, literatures and resources from open databases related to HDL-C were reviewed to screen AS targets. Then, PPI network construction and pathway enrichment analysis were used to explore the potential mechanism of resistance to AS. The results of KEGG analysis showed that Lipid and atherosclerosis, Cytokine-cytokine receptor interaction, Cholesterol metabolism, TNF signaling pathway, and PPAR signaling pathway are the core regulatory pathways after the elimination of unrelated findings. Based on the above results, we attempted to build the connection between increased HDL-C/anti-AS and lipid metabolism and the resolution of inflammation. We calculated Nim in the PPI network for AS disease. The above results were sorted out and combined, and then related literature reading was carried out.

According to the above method, we obtained three key protein genes: Apolipoprotein A1 (*APOA1*), Lipase C (*LIPC*), and Cholesteryl Ester Transfer Protein (*CETP*). *APOA1* is a necessary component of HDL particles. It plays a key role in the biosynthesis of HDL, cholesterol transport, and RCT [52]. It also has anti-inflammatory, anti-atherogenic, anti-apoptotic, and anti-thrombotic properties. *APOA1* helps stabilize vulnerable plaque by removing cholesterol from atherosclerotic plaque and reducing the damage caused by lipids [53]. Previous studies have shown that serum *APOA1* is positively correlated with HDL-C in the Chinese population [54]. *APOA1* (M148A) mutation may interfere with HDL remodeling. Low-density lipoprotein cholesterol (LDL-C) levels were reduced in mice with HDL particles carrying *APOA1* (Y192A) [55]. *APOA1* and important *APOA1* mutations are still being studied and developed for the treatment of CVD, AS, and other diseases. *CETP* promotes the transfer of cholesterol esters from HDL to very low-density lipoproteins and low-density lipoprotein (LDL). Therefore, *CETP* inhibitors were developed to increase HDL-C levels and lower LDL-C levels to prevent CVD. Unfortunately, recent large clinical trials have proven disappointing results. However, researchers are still full of confidence in its potential protective effect against the risk of CVD and diabetes [56, 57]. Liver lipase is a lipolytic enzyme involved in plasma lipoprotein metabolism, especially HDL metabolism. Studies have identified common variants of the hepatic lipase gene associated with HDL cholesterol [58]. According to reports, HDL is mainly participating in RCT. In addition, HDL also has several new functions such as inhibiting endothelial inflammation, promoting endothelial production of NO and prostacyclin, as well as isolating and transporting amyloid-producing proteins, oxidized lipids, and lipids derived from exogenous pathogens [59]. In subjects with extremely high HDL-C levels, rare or common variants of *CETP* and common variants of *LIPC* are often found [60]. This suggests expression levels of these genes may have an important effect on HDL.

Molecular docking is then performed to screen candidates with high therapeutic potential. Based on the analysis, a small molecule with high affinity, Genistein, was selected. Genistein is a 7-hydroxyisoflavone with the molecular formula of C15H10O5 and a molecular weight of 270.24 g/mol. It belongs to a class of isoflavone compounds, which mainly come from legumes. It has various biological activities such as lowering glucose, lowering lipids, antioxidants, anti-inflammatory, and anti-tumor [61]. It was found that Genistein may reduce AS via activating PPARγ-LXRα-ABCA1/ABCG1 pathway to enhance cholesterol effluence [62]. In this study, foam cells were induced in vitro to simulate the formation of foam cells in AS plaques. In our study, Genistein significantly up-regulated *APOA1* and *LIPC* levels in a dose-dependent manner. Although the regulation of *CETP* by Genistein is not obvious, this may be because there is no suitable PCR primer sequence at present. Based on our findings, we infer that Genistein may be a promising drug candidate in the treatment of AS. It can effectively regulate and raise therapeutic targets associated with HDL-C levels.

The advantage of this study is that the targets related to AS are obtained by integrating multiple databases. Then, the importance of nodes and pathway enrichment were calculated by the formula, and the key therapeutic targets of HDL-C for the treatment of AS were finally obtained through literature collection: *APOA1*, *LIPC*, and *CETP*. The most suitable potential natural product, Genistein, was found through molecular docking simulation, and the Genistein and the target were verified by in vitro experiments. It is an integrated multidisciplinary approach to establishing a viable anti-AS drug discovery process. To the present knowledge, no published study has so comprehensively screened and validated a natural product that can raise HDL-C levels as an anti-AS therapeutic target. Of course, there are some limitations to this study. First, we only verified the relationship between possible targets and Genistein through computer simulations and in vitro experiments, not in vivo experiments. Second, we did not compare Genistein with existing treatments, nor did we conduct a combination intervention to further evaluate its effects on HDL-C and AS. Third, Genistein is present in a variety of plants, and the best source for clinical use has not been identified. Therefore, the potential of Genistein to increase the level of HDL-C, as well as the mechanism and effect of the treatment of AS need to be further confirmed in vivo and in vitro. Experiments are also needed to determine the best source of Genistein.

Although this study gives insights into treating AS by increasing HDL-C levels, further research and validation are needed. In future studies, we may consider conducting long-term population-cohort studies of AS to track exposure, HDL levels, and the progression of associated diseases in a large number of participants. This helps us to more fully assess the potential role of HDL-C in disease progression, as well as its interaction with other biomarkers. In addition, animal experiments are also a powerful way to verify the association between our found targets and HDL-C, which can further elucidate the relevant mechanisms regulating HDL-C by simulating atherosclerosis models. This will provide us with a more comprehensive and in-depth understanding of the mechanism of action and potential natural products in the treatment of AS, and provide an important theoretical basis and a new way for the development of future treatment strategies and new drugs for AS.

## Conclusions

In summary, through bioinformatics combined with molecular docking and in vitro experimental verification, this study explored the possible potential small molecules of natural products and their mechanism of action in the treatment of AS by increasing HDL-C levels. GEO and PPI network analysis is helpful to identify several key target genes related to AS and to screen small molecules of TCM for the treatment of AS using molecular docking techniques. In addition, we confirmed that small molecules have significant regulatory effects on related protein genes through in vitro experiments. A series of comprehensive analysis methods established in this study is expected to provide a way of thinking and powerful technical support for the treatment of AS and the development of novel molecules of natural products.

### Supplementary Information

**Additional file 1: Table S1.** Primer sequences used for qRT-PCR.

**Additional file 2: Table S2.** The six potential TCM small molecules were searched from HIT 2.0.

## Data Availability

All data analyzed during this study are included in the websites mentioned above.

## Abbreviations

LDL-C: Low-density lipoprotein cholesterol
LDL: Low-density lipoprotein
AS: Atherosclerosis
CVD: Cardiovascular diseases
RCT: Reverse cholesterol transport
HDL-C: High-density lipoprotein cholesterol
HDL: High-density lipoprotein
TCM: Traditional Chinese Medicine
GEO: Gene Expression Omnibus
PPI: Protein–protein interaction
Nim: Node importance
GO: Gene ontology
KEGG: Kyoto Encyclopedia of genes and genomes
DMEM: Dulbecco’s modified Eagle’s medium
ox-LDL: Oxidized low-density lipoprotein
RT-qPCR: Reverse transcription‑quantitative polymerase chain reaction
*APOA1*: Apolipoprotein A1
*CETP*: Cholesterol ester transfer protein
*LIPC*: Lipase C
