# Supplementary material for: Identifying the natural products in the treatment of atherosclerosis by increasing HDL-C level based on bioinformatics analysis, molecular docking, and in vitro experiment
Source: J Transl Med. 2023 Dec 19;21:920. doi: 10.1186/s12967-023-04755-7 (PMC10729509; doi:10.1186/s12967-023-04755-7)
Supplement: Supplementary file 1 — Additional file 1: Table S1. Primer sequences used for qRT-PCR. [file 12967_2023_4755_MOESM1_ESM.docx]

**Tab. S1** Primer sequences used for qRT-PCR

| Gene | Forward primer | Reverse primer |
| --- | --- | --- |
| GAPDH | AGGTCGGTGTGAACGGATTTG | TGTAGACCATGTAGTTGAGGTCA |
| *APOA1* | CTTGGCACGTATGGCAGCA | CCAGAAGTCCCGAGTCAATGG |
| *LIPC* | GGAAATCCCCTCCAAATCTCCA | GCTGAGGTCTGAGACGACAG |
| *CETP* | CTTGTCCATCGCCACCAGCC | AGGGAGTGGAAGACTTGCTCGGA |
