# Supplementary material for: Identifying the natural products in the treatment of atherosclerosis by increasing HDL-C level based on bioinformatics analysis, molecular docking, and in vitro experiment
Source: J Transl Med. 2023 Dec 19;21:920. doi: 10.1186/s12967-023-04755-7 (PMC10729509; doi:10.1186/s12967-023-04755-7)
Supplement: Supplementary file 2 — Additional file 2: Table S2. The six potential TCM small molecules were searched from HIT 2.0. [file 12967_2023_4755_MOESM2_ESM.docx]

**Tab. S2** The six potential TCM small molecules were searched from HIT 2.0

| HIT ID | Pubchem Name | Pubchem CID | Molecular Formula | CAS | Molecular Weight (g/mol) |
| --- | --- | --- | --- | --- | --- |
| C0055 | Estradiol | 5757 | C18H24O2 | 50-28-2 | 272.40 |
| C0101 | Genistein | 5280961 | C15H10O5 | 446-72-0 | 270.24 |
| C0383 | Salicylic Acid | 338 | C7H6O3 | 69-72-7 | 138.12 |
| C0554 | Cholesterol | 5997 | C27H46O | 57-88-5 | 386.70 |
| C0569 | Sucrose | 5988 | C12H22O11 | 57-50-1 | 342.30 |
| C0657 | 27-Hydroxycholesterol | 123976 | C27H46O2 | 20380-11-4 | 402.70 |
